# Female Pelvic Floor Disorders in Northern India: Uncommon or Underreported?

**DOI:** 10.7759/cureus.74203

**Published:** 2024-11-22

**Authors:** Ankur Mittal, Arunima Saini, Vikas K Panwar, S Chezhian, Yogesh Bahurupi, Mahendra Gehlot

**Affiliations:** 1 Urology, All India Institute of Medical Sciences, Rishikesh, Rishikesh, IND; 2 Fellow Female Pelvic Medicine & Reconstructive Surgery, Department of Urology, All India Institute of Medical Sciences, Rishikesh, Rishikesh, IND; 3 General Surgery, All India Institute of Medical Sciences, Rishikesh, Rishikesh, IND; 4 Community and Family Medicine, All India Institute of Medical Sciences, Rishikesh, Rishikesh, IND; 5 Social and Preventive Medicine, All India Institute of Medical Sciences, Rishikesh, Rishikesh, IND

**Keywords:** colorectal, disorders, dysfunction, pelvic floor, urinary

## Abstract

Context: Pelvic floor disorders (PFD) have been on the rise, with an overall prevalence of 11%-35.5% globally. They develop due to various factors like increasing number of deliveries and increasing age, leading to progressive weakening of the soft tissues and pelvic support system. They encompass a variety of symptoms involving the urinary, uterovaginal, and colorectal compartments. Patients either have symptoms isolated to single or multiple compartments concomitantly.

Aims: We found very limited literature denoting the exact prevalence of PFD in India. Due to the hesitant nature of females in a developing country like India and having a social taboo disclosing the common illnesses of females, these disorders are commonly hidden by females. We intended to know the prevalence of PFD in the state of Uttarakhand and create awareness in patients.

Settings and design: It was an intramurally funded project conducted over a duration of two years.

Methods and material: In the Doiwala block of Dehradun district, there are five PHCs. These 700 samples were proportionally allocated in these five PHCs as per the PPS method. All women 18 years of age and above (Doiwala Block), women residing in field areas, and those willing to participate were included in the study.

Statistical analysis: The data was analyzed using statistical and inferential statistics. Data analysis was done using IBM Corp. Released 2015. IBM SPSS Statistics for Windows, Version 23.0. Armonk, NY: IBM Corp.

Results and conclusions: We reported a higher prevalence of 56% in females of Uttarakhand, with increasing parity being the direct cause. Also, we found the majority of females with PFD to have them in moderate severity. Also, 89.6% of females had symptoms in all three compartments. We compared the two questionaries and concluded that PFDI 20 was better and more detailed than PFQI-7.

## Introduction

Women usually hesitate to disclose their problems related to pelvic floor disorders (PFD) due to associated social stigma and lack of access to services. Underreporting has also been attributed to the significant financial cost of its management and the belief that incontinence is a normal part of aging and not a treatable condition. Studies have shown that 11%-35.5% of women suffer from PFD globally [1,2]. PFDs are associated with an increasing number of deliveries and increasing age, leading to progressive weakening of the soft tissues and pelvic support system [3,4]. Most commonly, the age above 55 years has been seen to be associated with increased weakness resulting in PFD [5,6].

PFDs are troublesome as they impact the woman’s day-to-day activities and add to distress and discomfort. The females from developing countries try to avoid the tendency and bear the ailment till it becomes unbearable for them, and in the end, they have no option but to consult the doctor and seek treatment [7,8]. Despite the poor quality of life of women with pelvic floor disorders (urinary incontinence, pelvic organ prolapses, and dysfunctional voiding), they remain underreported and undertreated. This makes assessment of the prevalence of these disorders difficult. There is a scarcity of literature regarding female pelvic disorders in the Indian population. The present study is planned to be conducted in the plains and foothills areas of Uttarakhand. Apart from studying the prevalence, associated risk factors, and impact of pelvic floor disorders on QOL in the Indian community, the scope is to create awareness among women regarding the same.

## Materials and methods

It was a community-based cross-sectional study done in the field practice area on all women above 18 years of age using a systematic random sampling technique.

Inclusion criteria 

All the women are 18 years of age and above (Doiwala Block). All the women residing in the field practice area. All the women who were willing to participate.

Exclusion criteria 

Women with acute illness, recent abdominal surgery, musculoskeletal issues, spinal cord injury, or cerebral palsy were excluded from the study.

Objectives

A) To study the prevalence of pelvic floor disorders in the Indian community. B) To study associated risk factors with pelvic floor disorders. C) To study the impact of pelvic floor disorders on quality of life.

The sample size calculation was done by: Prevalence of pelvic floor disorder (20%)* =700 approximately.

The total sample size is estimated by using the formula: 4pq/d2

Where n = sample size

P = Prevalence of pelvic floor disorder (20%)*

Q = 100-p =80%

D = Error in estimation (taking absolute error 5%)

The required sample size is calculated as: n = (4x0.20x0.80)/(0.05) 2 =256

Assuming 20% of non-respondents and applying a design effect of two, the sample size came out to be 620, which was rounded off to 700. We had a total sample size of 697 patients, which were proportionally allocated in these five PHCs of the Doiwala block of district Dehradun, as per the PPS method. The obtained sample size of each PHC again was proportionally allocated in their sub-centers by the PPS method. Targeted samples in each sub-center were selected by systematic random sampling to provide maximum representation of the sub-center.

The study tool consisted of a demographic schedule and PFDI-20 and PFIQ-7 questionnaires. The participants were approached in a field area and explained about the study creating awareness by a female counselor. After their consent, validated questionnaires were filled. Participants were also counseled about the available treatment options. All the information was collected, and a data Excel sheet (Redmond, USA) was prepared. The data was analyzed using statistical and inferential statistics. Data analysis was done using IBM Corp. Released 2015. IBM SPSS Statistics for Windows, Version 23.0. Armonk, NY: IBM Corp. Prevalence was reported with a 95% confidence interval. A chi-square test was used to determine the association between demographic factors, obstetric factors, and pelvic floor dysfunction. These factors were then analyzed using a multivariate logistic regression model to determine the contribution of these factors in the development of PFD.

## Results

The data was prepared in relation to the presence of pelvic floor disorders with various demographic variables. Taking into account two questionnaires, PFDI-20 and PFQI-7, the prevalence of pelvic floor disorders was calculated. It was found that the prevalence of pelvic floor disorders was 56.7% (395 of 697 patients).

A total of 41.5% (289 of 697) patients were illiterate, 29.3% (204 of 697) patients were educated between classes 1-10, while only 11.2% (78 of 697) patients were educated to undergraduate level. Among these, pelvic floor symptoms were found to be more common in illiterate patients (45%), followed by decreasing incidence in educated patients up to classes 1-10 (28.9%), 11-12 (12.7%), undergraduate (10.6%), and least common in postgraduates (2.8%). It signified the effect of educational awareness towards pelvic floor disorders. Although, the difference was not statistically significant (p=0.128).

Patients were subdivided based on their occupation into a housewife, student, white-collar job, skilled, semiskilled, and unskilled. It was found that the maximum patient population residing in the areas studied were housewives (83.5%). The majority (85.8%) of the patients who had pelvic floor disorders were housewives, followed by skilled workers (6.1%), white collar jobs (2%), unskilled workers (3.5%), and students (0%). However, no statistical difference was found between the patients with and without pelvic floor disorders based on occupation (p=0.381).

Our study population comprised 77.4% married candidates, followed by 13.5% divorced/widowed and 8.9% unmarried females above 18 years of age. 79.2% of patients with pelvic floor disorders were married, and 6.1% were unmarried. A significant increase in pelvic floor disorders was seen in the married population (p=0.009). Also, the age of marriage contributed to the prevalence of pelvic floor disorders. In females with the age of marriage <18 years, 7.3% had cesarean sections, while in >18 years, 9.6% had cesarean sections. 92.6% of women with age at marriage <18 years had vaginal delivery, while it was 90.4% in >18 years. This difference was not statistically significant (p=0.4). Pelvic floor disorders were greater (77.4%) with increasing age at marriage (>18 years). Marriages less than 18 years showed lesser prevalence of pelvic floor disorders (22.6%). Cesarean section could be the confounding factor that resulted in a significant difference in pelvic floor disorders in <18 years. Although this significant difference does not have practical implications in the Indian community, teenage marriage (<18 years) is not permitted by Indian law. 87.4% of the symptomatic patients with pelvic floor disorders had vaginal deliveries, while 10.4% had undergone cesarean section. A small proportion (2.2%) of patients with pelvic floor disorders had both cesarean sections and normal vaginal delivery. Although the difference was not statistically significant, a greater proportion of pelvic floor disorders were seen to occur in patients who had undergone vaginal deliveries (p=0.636).

A statistically significant increased prevalence of pelvic floor disorders was seen with increasing parity (p=0.001). The parity of the patients was categorized into nulliparous, primiparous, multiparous, and grand multiparous. The major bulk of pelvic floor disorders were seen in multiparous patients (76.7%), followed by primiparous (8.4%), nulliparous (8.1%), and grand multiparous patients (6.8%) (Table 1).

**Table 1:** Relation of pelvic floor disorders with demographic parameters (*: statistically significant p-value below 0.05)

Parameters		Pelvic floor disorder		Total (%)	p-value
		Present	Absent		
Educational status	No schooling	178 (45.1%)	111 (36.8%)	289 (41.5%)	0.128
	1-10	114 (28.9%)	90 (29.8%)	204 (29.3%)	
	11-12	50 (12.7%)	50 (16.6%)	100 (14.3%)	
	Undergraduate	42 (10.6%)	36 (11.9%)	78 (11.2%)	
	Postgraduate	11 (2.8%)	15 (5.0%)	26 (3.7%)	
Occupation	Housewife	339 (85.8%)	243 (80%)	582 (83.5%)	0.381
	Student	0 (0%)	0 (0%)	0 (0%)	
	White-collar job	8 (2%)	3 (1%)	11 (1.6%)	
	Skilled	24 (6.1%)	35 (11.6%)	59 (8.5%)	
	Semi-skilled	10 (2.5%)	12 (4%)	22 (3.2%)	
	Unskilled	14 (3.5%)	9 (3%)	23 (3.2%)	
Marital status	Unmarried	24 (6.1%)	38 (12.6%)	62 (8.9%)	0.009*
	Married	313 (79.2%)	228 (75.5%)	541 (77.6%)	
	Divorced/Widow	58 (14.7%)	36 (11.9%)	94 (13.5%)	
Age at marriage	<18	83 (22.6%)	39 (14.7%)	122 (19.3%)	0.012*
	>=18	284 (77.4%)	227 (85.3%)	511 (80.7%)	
Mode of delivery	Vaginal	312 (87.4%)	229 (89.8%)	541 (88.4%)	0.636
	Caesarean	37 (10.4%)	22 (8.6%)	59 (9.6%)	
	Both	8 (2.2%)	4 (1.6%)	12 (2%)	
Parity	Nulliparous	32 (8.1%)	48 (15.9%)	80 (11.4%)	0.001*
	Primiparous	33 (8.4%)	39 (12.9%)	72 (10.3%)	
	Multiparous	303 (76.7%)	199 (65.9%)	502 (72%)	
	Grand multiparous	27 (6.8%)	16 (5.3%)	43 (6.2%)	
Total		395 (56.7%)	302 (43.3%)	697	

We used two questionnaires, PFDI 20 (Pelvic floor distress inventory) and PFIQ 7. PFDI 20 comprises 20 questions divided into three subgroups: POPDI-6, CRADI-8, and UDI-6, focusing on questions specific to pelvic organ prolapse, colorectal, and urinary symptoms in each. PFIQ 7 consists of seven questions asked to assess prolapse, colorectal, and urinary symptoms classified as POPIQ-7, CRAIQ-7, and UIQ-7. The presence of any one of the symptoms denotes pelvic floor disorder. That was further graded into mild (1-33), moderate (34-66), and severe (67-100) symptoms based on the scoring. A total score was calculated by the three scores and again divided into 0-100, 100-200, and 200-300.

Table 2 illustrates the effect of age on an individual subset of pelvic floor symptoms. An increase in mean age was noted in patients with increasing severity of pelvic organ prolapse and colorectal and urinary symptoms. The mean age of patients with mild symptoms in the POPDI 6 score was 42.19±16.0 years, moderate symptoms were 41.13±14.8 years, and severe symptoms were 48.24±13.1 years. Similarly, an increase in mean age was seen in colorectal symptoms (CRADI -8 score) from 42.19±16.0 years in patients with mild symptoms to 48.24±13.1 years in patients with severe symptoms. Urinary symptoms were also associated with an increase in mean age from 42.28±15.9 years in patients with mild symptoms to 42.28±15.9 years in patients with severe symptoms. However, these were not statistically significant on ANOVA analysis. Maximum symptoms in urinary, colorectal, and pelvic organ prolapse were mild, followed by moderate symptoms and severe symptoms in decreasing order of prevalence. However, the increasing age supports the increase in the symptoms of the patient with respect to all 3 compartments, urinary, pelvic organ prolapse, and colorectal, due to normal physiological changes of loss of collagen and soft tissue and decreased vascularity due to hormonal withdrawal in the peri and postmenopausal age groups.

**Table 2:** Grading severity of pelvic floor disorders with age using PFDI 20 and PFQI scoring (#: ANOVA test)

Subset	Asymptomatic 0	Mild symptoms 1-33	Moderate symptoms 34-66		Severe symptoms 67-100	p-value
POPDI-6	28	190	160		17	0.305#
	43.89±17.7 (37.05-50.74)	42.19±16.0 (39.89-44.48)	41.13±14.8 (38.81-43.45)		48.24±13.1 (41.5-54.9)	
CRADI-8	28	190	160		17	0.305#
	43.89±17.7 (37.05-50.74)	42.19±16.0 (39.89-44.48)	41.13±14.8 (38.81-43.45)		48.24±13.1 (41.5-54.9)	
UDI-6	11	128	188		68	0.264#
	42.73±11.55 (34.97-50.49)	42.28±15.9 (39.5-45.07)	40.88±15.19 (38.7-43.0)		45.26±16.4 (41.3-49.23)	
POPQI	110	178	98		9	0.459#
	41.64±15.1 (38.8-44.5)	41.22±15.9 (38.86-43.58)	44.19±15.54 (41-47.3)		44.22±14.34 (33.2-55.25)	
CRAQI	219	137	25		14	0.661#
	42.78±15.1 (40.76-44.80)	41.6±16.5 (38.8-44.39)	41.92±16.67 (35-48.8)		37.8±11.2 (31.4-44.3)	
UQI	298	77	15		5	0.292#
		42.6±15.4 (40.8-44.36)	41.96±16.8 (38.1-45.79)	37.0±12.75 (30-44.13)	32.6±3.71 (27.99-37.21)	

In the PFIQ questionnaire, the majority of the patients in POPQI had mild symptoms (178/395), while CRAQI (219/395) and UQI (298/295) had no symptoms (Table 2).

In our study, we correlated the severity of pelvic floor symptoms with increasing parity. Parity was subdivided into nulliparous, primiparous, multiparous, and grand multiparous categories. The majority of patients with pelvic organ prolapse, colorectal symptoms, and urinary symptoms were multiparous (303/395) in both scoring systems. Also, our study revealed decreasing symptoms in grand multipara patients, which was probably due to falling awareness and the low socioeconomic group in the patient population being studied. Also, the bulk of patients were in the multiparous category, making it difficult to comment on grand multiparous patients. A significant increase in colorectal symptoms was noticed with increasing parity, as demonstrated by the CRAQI score (p=0.098). Also, an increase in the mean age of patients was seen in grand multiparous patients as compared to nulliparous and primiparous patients (Table 3).

**Table 3:** Effect of parity on pelvic floor disorders (#: ANOVA test; *: Statistically significant p-value below 0.05)

Subset	Nulliparous	Primiparous	Multiparous	Grand multiparous	p-value
POPDI-6	32	33	303	27	0.677#
	27.38±17.8 (20.9-33.8)	26.68±22 (18.87-34.48)	30.34±19.98 (28-32.6)	30.4± 19.6 (22.65-38.15)	
CRADI-8	32	33	303	27	0.275#
	28.96±16 (23.18-34.74)	29.87±15.6 (24.32-35.42)	33.72±17.69 (31.72-35.72)	35.66±18.59 (28.3-43.01)	
UDI-6	32	33	303	27	0.317#
	42.26±20 (35-49.5)	51.55±20.4 (44.28-58.8)	46.77±21.7 (44.31-49.23)	43.82±20.26 (35.8-51.83)	
POPQI	32	33	303	27	0.574#
	5.36±12.6 (.79-9.93)	3.17±8.95 (0.00-6.35)	6.20±15.96 (4.4-8.01)	3.35±8.73 (10-6.81)	
CRAQI	32	33	303	27	0.098*#
	12.2±18.29 (5.61-18.8)	5.77±12.87 (1.21-10.34)	11.29±19.4 (9.08-13.49)	18.52±27.62 (7.59-29.44)	
UQI	32	33	303	27	0.843#
	23±27.9 (13.1-33.3)	21.64±20.6 (14.3-28.98)	24.15±22.8 (21.57-26.73)	20.8±19 (13.26-28.33)	

We enrolled a total of 697 females (three dropouts excluded), and the prevalence of pelvic floor disorders was found to be 56.5% (394/697). Among these, only 6.1% of patients (24/394) had a single symptom on PFDI scoring and 39% (121/310) on PFQI scoring. 79.2% of patients with isolated compartment symptoms were urinary in both PFDI and PFQI scoring, followed by colorectal and pelvic organ prolapse symptoms. Multiple symptoms involving all three compartments were seen in 89.6% of patients (PFDI) and 65% of patients (PFQI). A very small percentage of patients had isolated urinary and prolapse symptoms or prolapse and colorectal symptoms. The PFDI 20 score was good in interrogating patients with each compartment-specific question and finding co-existing adjacent compartment involvement (Table 4).

**Table 4:** Division of patients according to symptoms

Number of symptoms	Symptoms	PFDI-20, n (%)	PFQI-7, n (%)
Single	Pelvic organs prolapse	2 (8.3%)	12 (9.9%)
	Urinary	19 (79.2%)	96 (79.3%)
	Colorectal	3 (12.5%)	13 (10.7%)
	Total	24 (6.1%)	121 (39%)
Multiple	Prolapse+ Urinary	4 (1%)	21 (6.8%)
	Prolapse+ Colorectal	4 (1%)	0(0%)
	Urinary + Colorectal	56 (14.2%)	97(31.3%)
	Prolapse+Urinary+Colorectal	353 (89.6%)	65 (20.0%)
Total symptomatic patients		394 (56.5%)	310 (44.5%)

A total of 27.3% of patients had mild prolapse and urinary symptoms, 23% of patients had moderate prolapse and urinary symptoms, while only 2.4% of patients had severe prolapse and urinary symptoms. Colorectal symptoms were moderate in 27%, mild in 18.4%, and severe in 9.8% of patients. The total PFDI 20 score was in the moderate category in 30.8% (215/697) patients followed by the mild category in 23.8% (166/697) patients. Only 11% (77/697), 25.5% (178/697) and 19.7%.

A total of 137/697 patients were in the mild pelvic organ prolapse and urinary symptoms and colorectal symptoms category, while the majority of patients were asymptomatic (86%, 59.1%, and 74.4% for pelvic organ prolapse, urinary, and colorectal symptoms, respectively). As per the total PFIQ score, 39.9% of patients were in the mild symptom category, while in the PFDI score, 30.8% of patients were in the moderate symptom category, highlighting the better symptom interrogation and picking up severity of disease with the PFDI 20 questionnaire in contrast to the PFQI-7 questionnaire (Table 5). 

**Table 5:** Prevalence of pelvic floor disorders in community

Questionnaire	Subset	Categorization				Total score			
		0	1-33	34-66	67-100	0	1-100	101-200	201-300
PFDI-20	POPDI-6	330 (47.3%)	190 (27.3%)	160 (23%)	17 (2.4%)	303 (43.5%)	166 (23.8%)	215 (30.8%)	13 (1.9%)
	CRADI-8	313 (44.9%)	128 (18.4%)	188 (27%)	68 (9.8%)				
	UDI-6	330 (47.3%)	190 (27.3%)	160 (23%)	17 (2.4%)				
PFIQ-7	POPIQ-7	600 (86.1%)	77 (11%)	15 (2.2%)	5 (0.7%)	387 (55.5%)	278 (39.9%)	30 (4.3%)	2 (0.3%)
	CRAIQ-7	521 (74.4%)	137 (19.7%)	25 (3.6%)	14 (2%)				
	UIQ-7	412 (59.1%)	178 (25.5%)	98 (14.1%)	9 (1.3%)				

## Discussion

Pelvic floor distress inventory (PFDI-20) is a patient-reported outcome measure used in clinical practice for the assessment of pelvic floor dysfunction. This has been advised as a grade A recommendation by the International Consultation on Incontinence (ICI) [8]. Our study showed a very high prevalence of pelvic floor disorders in the Uttarakhand population. Limited Indian studies are available to show the prevalence of pelvic floor disorders. The presence of even a single compartment involvement was counted, resulting in an overall rate of 56%. As the general population of Uttarakhand location is involved in moderate to heavy household work, accounting for its location in a hilly area, this might account for a higher incidence of pelvic floor disorders in our study. A study from South India by Vijayalaksmi R et al. on postpartum women between 3 months and one year showed a similar prevalence of 54.7%, although the sample size was only 424 women [9]. Moreover, the selected target population was only postpartum patients, while we went from house to house and surveyed each female in our study irrespective of age, parity, and occupation, limiting ourselves within the inclusion and exclusion criteria defined for the study. Another study by Bhamini Krishna Rao et al. reported the prevalence of pelvic floor dysfunction to be 21%, done on 1256 women at six centers of rural maternity and child welfare (RWCW) in Karnataka [10].

The study by Nygaard et al. reported the prevalence of at least one pelvic floor dysfunction in women to be 23.7% and only 2.9% of the women having pelvic organ prolapse [11]. Due to multiple factors like the need for multiple children, strenuous physical activity, and household jobs in comparison to the normal plain area population, the hesitant attitude of women, and presuming these conditions to be a part of a normal lifestyle, these conditions are ignored and not brought to physician notice till they significantly hinder the day-to-day activities. 

It is uncommon to find symptoms isolated to a single compartment, either urinary, colorectal, or prolapse. Single-compartment symptoms may be more profound despite the presence of multiple-compartment involvement. In our study, we found that the incidence of prolapse was 8.3% (9.3% by PFQI score) and colorectal symptoms were 12.5% (10.7% by PFQI score), while urinary symptoms (difficulty in urination, lower urinary tract symptoms, incontinence, voiding issues) were much more prevalent, up to the tune of 79.2%. A total of 121/697 (39%) patients showed single-compartment involvement symptoms (PFIQ 7 score). This was supported by a study by Vijayalaksmi R et al., with 82.4% of patients having urinary incontinence, 50% having bowel incontinence, and 1.4% having pelvic organ prolapse. Another Indian study has reported the incidence of urinary incontinence to be 19.02% and pelvic organ prolapse to be 1.99%, much lower than our study results owing to the different geographical areas [12].

Zeleke et al. (12) conducted a study in Australia and found that among 1517 women, 47.2% of women had one or more pelvic floor disorders, and 6.8% had POP. Among the women with POP, 53.4% had UI. In our study, 89.6% of patients had all three compartment symptoms, while both prolapse and urinary, urinary, and colorectal accounted for 6.8% and 31.3% of patients, respectively. A study on a larger patient population of 3432 women by Dheresa et al. showed 20.5% of patients with at least one type of pelvic floor disorder and 49.6% reported two or more pelvic floor disorders [13]. The most common pelvic floor disorders included an overactive bladder (15.5%), pelvic organ prolapse (9.5%), stress urinary incontinence (8.3%), and anal incontinence (1.9%). The numbers vary with the increasing patient population under study, and the greater the number, the lesser the chances of error.

An interesting study by David Y et al. was done to evaluate pelvic floor distress symptoms in postpartum patients at 24 h and 3 months postdelivery period [14]. It showed that the colorectal and anal distress symptoms increased to the tune of 31.5%. The study concluded that the presence of episiotomy and birth weight were not found to be significantly associated with any of the PFD items. At the three-month interval follow-up, the colorectal symptoms decreased to 20.7% in patients, and pelvic organ prolapse symptoms and urinary symptoms were 12.8% and 15.8%, respectively.

In our study, we did not include antenatal and postpartum patients, as the symptoms are transiently worsened due to the compression effect of the growing fetus and hormonal changes.

The direct relationship between increasing parity and pelvic floor disorders has been seen in our study. The major bulk of patients were multiparous. Interestingly, the significance was noticed with colorectal symptoms, as demonstrated by the CRAQI score. A previous study by Zeleke et al. showed the maximum number of patients (52.1%) in parity of 3 and above and the rest (45.3%, 34.6%) with parity 1-2 and zero, respectively. They also showed the increased correlation of at least one pelvic floor disorder in obese patients. Bhamini Krishna Rao et al. also showed a higher prevalence of pelvic floor disorders in women having 2 or 3 children and in those who delivered vaginally. Dheresa et al. demonstrated a twofold increase in pelvic floor disorders in women with vaginal delivery and the effect of increasing parity on PFD.

In our study, we studied the various factors and their association with the pelvic floor disorders. We found a direct correlation with increasing parity and an inverse correlation with age at marriage. Whereas, other factors like the age, educational status of women, occupation, and menopausal status were not seen to be associated with increased prevalence of PFD. Also, we classified the symptoms of various compartments into mild, moderate, and severe categories using two scales. We concluded that the PFDI-20 was an elaborative questionnaire helping us know better about patients symptoms and degree of ailment. A study by Mattson et al. also showed a lesser response rate to the PFQI-7 questionnaire, the reasons for which could not be conclusive. 30.8% of patients had moderate symptoms, 23.8% had mild symptoms, and only 1.9% had severe symptoms [15]. The remaining 43.5% were asymptomatic patients. This shows the higher prevalence of PFD in this belt of Uttarakhand.

Various limitations in our study were the paucity of literature showing the prevalence of PFD in the Indian population. Also, we labeled the patient's severity only by questionnaires, and examination was not a part of our study, although examining patients would have added to our results. We could not diagnose patients with pelvic floor dysfunction, neurogenic or myogenic etiology. Another limitation of the study was the possibility of c-sections as a confounding factor for achieving statistical significance for comparison between the age of marriage less than and above 18 years of age. Also, we could not get information about details of urinary symptoms like stress or urgency urinary incontinence.

## Conclusions

Pelvic floor disorders in females are common but hidden. Its prevalence varies with the area, ethnicity, and population being studied. We reported a higher prevalence of 56% in females of Uttarakhand, with increasing parity being the direct cause. Also, we found the majority of females with PFD to have them in moderate severity. We compared the two questionnaires and concluded that PFDI-20 was a better and more detailed one as compared to PFQI-7. We intended to create awareness in the population studied and guide the patients to get themselves treated for these treatable causes, thereby stepping ahead towards improved quality of life in the female population of a developing country like ours.
